# Mitochondrial Deformation During the Cardiac Mechanical Cycle

**DOI:** 10.1002/ar.23917

**Published:** 2018-10-10

**Authors:** E. A. Rog‐Zielinska, E. T. O'Toole, A. Hoenger, P. Kohl

**Affiliations:** ^1^ Institute for Experimental Cardiovascular Medicine, University Heart Center Freiburg, Bad Krozingen, and Faculty of Medicine University of Freiburg Freiburg Germany; ^2^ Department of Molecular, Cellular and Developmental Biology University of Colorado at Boulder Boulder Colorado USA

**Keywords:** mitochondria, heart, mechanosensitivity, electron tomography, sarcoplasmic reticulum

## Abstract

Cardiomyocytes both cause and experience continual cyclic deformation. The exact effects of this deformation on the properties of intracellular organelles are not well characterized, although they are likely to be relevant for cardiomyocyte responses to active and passive changes in their mechanical environment. In the present study we provide three‐dimensional ultrastructural evidence for mechanically induced mitochondrial deformation in rabbit ventricular cardiomyocytes over a range of sarcomere lengths representing myocardial tissue stretch, an unloaded “slack” state, and contracture. We also show structural indications for interaction of mitochondria with one another, as well as with other intracellular elements such as microtubules, sarcoplasmic reticulum and T‐tubules. The data presented here help to contextualize recent reports on the mechanosensitivity and cell‐wide connectivity of the mitochondrial network and provide a structural framework that may aide interpretation of mechanically‐regulated molecular signaling in cardiac cells. Anat Rec, 302:146–152, 2019. © 2018 The Authors. *The Anatomical Record* published by Wiley Periodicals, Inc. on behalf of American Association of Anatomists.

As the heart beats, cardiomyocytes undergo significant phasic deformations with up to ±10% changes in length, compared to their resting state. The three dimensional (3D) organization of intracellular material will change, in this context, on a beat‐by‐beat basis—but how exactly is unknown.

Cardiomyocytes are densely packed, with “free cytosolic volume” as low as 4–7% (Kaasik et al., 2010), and neither the cell nor its membranous organelles such as mitochondria are presumed to change volume during the contractile cycle. Cardiomyocytes are enclosed by a deformable but nondistensible lipid bilayer that contains numerous surface folds and invaginations that unfold during strain (Kohl et al., 2003). This “spare membrane” addresses the otherwise problematic change in surface‐to‐volume ratio that would occur upon stretching an isovolumic “cylinder” (McNary et al., 2012; Pfeiffer et al., 2014). Intracellular organelles are arranged between strands of the contractile filament lattice, within a network of rigid and elastic nonsarcomeric cytoskeletal filaments. These organelles are influenced by deformations resulting from passive cardiomyocyte stretch and active shortening, a process aided by cytoskeletal filaments that bear and transmit mechanical loads throughout the cell (Bartolak‐Suki et al., 2017; Ingber, 2008; Robison et al., 2016). As shown for the surface sarcolemma (Kohl et al., 2003; Pfeiffer et al., 2014), some intracellular membranous structures, such as T‐tubules, contain “spare membrane” in the shape of caveolae (Burton et al., 2017).

Mitochondria are traditionally viewed as membrane‐enclosed prolate spheroids. They are amply present throughout cardiomyocytes, occupying ~30–40% of cell volume (Tsushima et al., 2018). They are sandwiched tightly between sarcomeres as well as clustered around the nucleus and in the subsarcolemmal space (Piquereau et al., 2013). Their main roles are energy metabolism and apoptotic pathway signaling. In cardiomyocytes, mitochondria have also been shown to be involved in Ca^2+^ signaling, potentially at time‐scales relevant for excitation‐contraction coupling. This mitochondrial Ca^2+^ signaling may be activated by local deformation (Belmonte & Morad, 2008).

Given the importance of mitochondria for cardiomyocyte function and their localization next to the contractile machinery, it seems natural to question whether, and if so how, their shape is affected by mechanical deformation of heart muscle, especially in the light of recent reports on mitochondrial mechanosensitivity (Liao et al., 2004; Bartolak‐Suki et al., 2017; Iribe et al., 2017; Schonleitner et al., 2017). The question arises as to whether mitochondria deform on a beat‐by‐beat basis, and if so, how they accommodate this with unchanged volume‐surface ratio. Based on transmitted light confocal line‐scanning and frequency domain analyses in isolated rat cardiomyocytes, it has been suggested that mitochondria do indeed shorten and widen during cell contraction (Yaniv et al., 2011), but due to the resolution of this technique, any potential deformation‐induced mismatch between volume and surface could not be characterized. In addition, interactions with the cytoskeleton, a key transmitter of mechanical stimuli within the cell, have also been shown to influence mitochondrial structure and function (Bartolak‐Suki et al., 2017), and it would be desirable to put this into a sarcomere‐length dependent context.

Here we investigate cardiomyocyte interfibrillar mitochondrial deformation as a function of sarcomere length using 3D electron tomography in intact rabbit left ventricular tissue, fixed in different mechanical states.

## MATERIALS AND METHODS

All investigations reported in this manuscript were ethically approved and conformed to the UK Home Office guidance on the Operation of Animals (Scientific Procedures) Act of 1986.

New Zealand white rabbit hearts (n = 6) were swiftly excised after euthanasia (pentobarbital overdose), Langendorff‐perfused with Krebs–Henseleit solution (containing [in mM]: NaCl 118, KCl 4.75, CaCl_2_ 2.5, NaHCO_3_ 24.8, MgSO_4_ 1.2, KH_2_PO_4_ 1.2, glucose 11, insulin 10 U L^−1^; bubbled with carbogen to achieve pH 7.4), and after a 5 min wash of the coronary circulation, the desired mechanical state was introduced. This involved: (i) contracture, induced by Na^+^‐for‐Li^+^ substitution in the perfusion buffer with concurrent caffeine application (10 mM), (ii) a resting “slack” state, achieved by cardioplegically arresting the heart using a high‐K^+^ (25 mM) version of Krebs–Henseleit solution, (iii) stretch, caused by intraventricular balloon inflation during cardioplegic arrest (as in ii). All solutions were controlled for iso‐osmolality (295–305 mOsm, Knauer AG, Berlin). As soon as the desired mechanical state was achieved (usually within 2 min), hearts were perfusion‐fixed with iso‐osmotic Karnovsky's fixative (3:1:1 mix of sodium cacodylate, paraformaldehyde, and glutaraldehyde; 300 mOsm; Solmedia Limited, Shrewsbury, UK; after (Karnovsky, 1964)). Tissue fragments were excised from the left ventricle and washed with 100 mM sodium cacodylate, post‐fixed in 1% OsO_4_ for 1 hr, dehydrated in graded acetone, and embedded in Epon‐Araldite resin. Semi‐thick (275 nm) sections were placed on formvar‐coated copper slot‐grids, post stained with 2% aqueous uranyl acetate and Reynold's lead citrate. Colloidal gold particles (15 nm) were added to both surfaces of the sections to serve as fiducial markers for tilt series alignment.

Preparations were imaged at the Boulder Laboratory for 3D Electron Microscopy of Cells (University of Colorado, Boulder, CO) using an intermediate voltage electron microscope (Tecnai TF30; FEI Company, now Thermo‐Fisher Scientific, Eindhoven, The Netherlands) operating at 300 kV. Images were captured on a 4 K × 4 K charge‐coupled device camera (UltraScan; Gatan, Pleasanton, CA) using the SerialEM software package (Ress et al., 1999). For tomographic imaging, the specimen holder was tilted from +60° to −60° at 1° intervals. For dual‐axis tilt series the specimen was rotated by 90° in the X–Y plane, before another +60° to −60° tilt series was taken. The images from each tilt‐series were aligned by fiducial marker tracking and back‐projected to generate two single full‐thickness reconstructed volumes (tomograms), which were then combined to generate a single high‐resolution 3D reconstruction of the original partial cell volume (Mastronarde, 1997; Mastronarde, 2005). Isotropic voxel size was (1.206 nm)^3^. All tomograms were processed and analyzed using IMOD software (Mastronarde, 2005), which was also used to generate 3D models of relevant structures (Kremer et al., 1996). Models were smoothed and meshed to obtain the final 3D representation, in which spatial relations of various cardiomyocyte substructures were quantified, as described before (Rog‐Zielinska et al., 2016).

## RESULTS

### Mechanically induced Changes in Mitochondrial Shape

**Figure 1:**
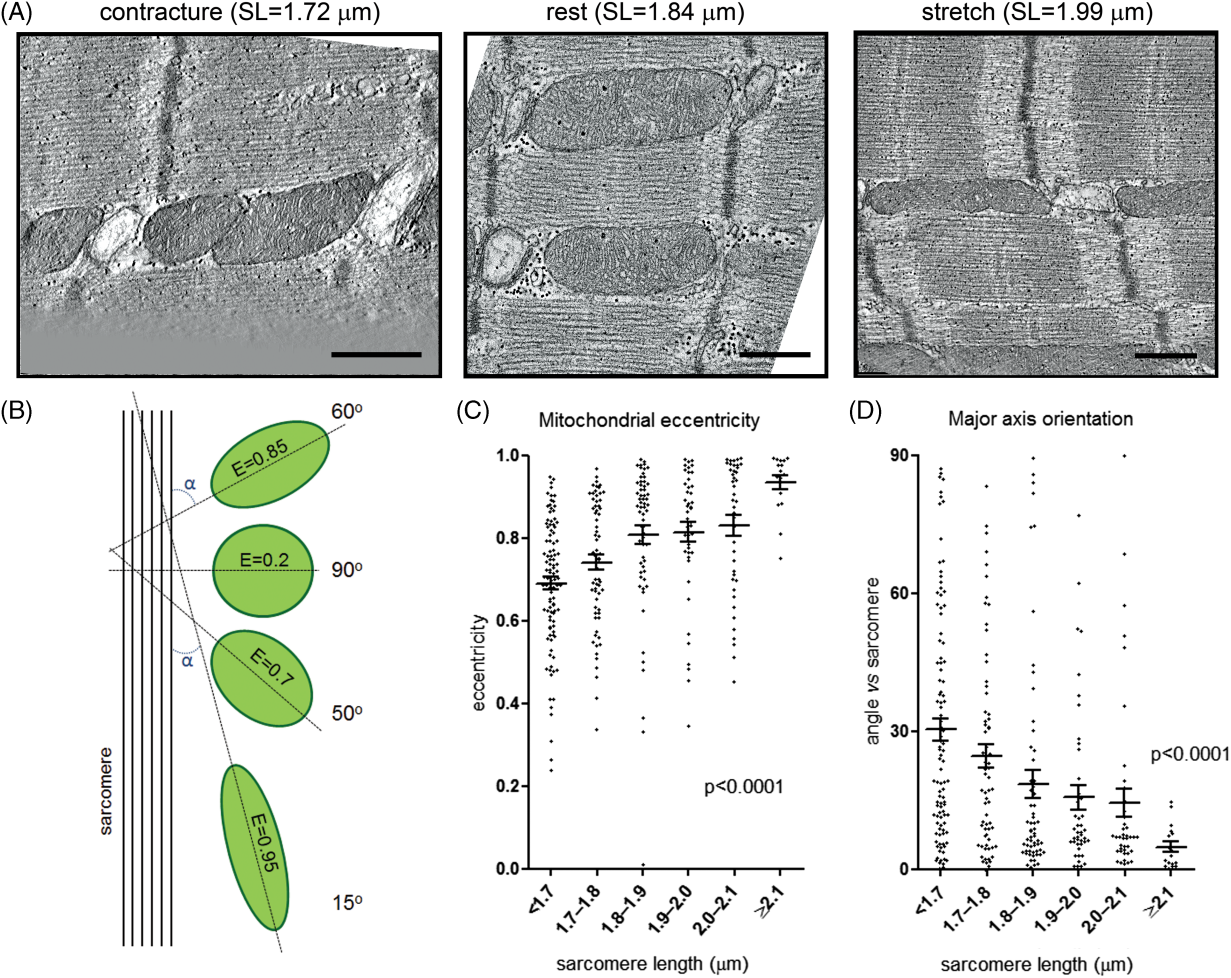
Mitochondrial shape changes with mechanical state of cardiomyocytes. (**A**) Representative electron tomographic micrographs of interfibrillar mitochondria in rabbit ventricular cardiomyocytes, fixed during contracture, at rest, and during stretch. (**B**) A schematic representation of the analysis performed to assess mitochondrial (green) shape (E = eccentricity) and main axis orientation in relation to contractile fibers (absolute value of minimum intersection angle, α between major axis and sarcomere plane). (**C**) Mitochondrial eccentricity is positively correlated with sarcomere length. (**D**) The absolute value of the minimum intersection angle between mitochondrial main axis and the sarcomere plane decreases with sarcomere length. Scale bars: 500 nm (in A); n = 17 to 103 mitochondria per group, N = 6 animals (in C and D), analysis with one‐way ANOVA.

Cross‐sectional shapes of interfibrillar mitochondria, analyzed in a plane parallel to contractile fibers, as well as the orientation of their major axis, are correlated with the mechanical state of the cell (Fig. 1). During stretch, mitochondria become elongated (i.e., exhibiting the highest eccentricity, here defined as *E*=$\frac{c}{a}$, where *c* is the distance from the center to the focus of the ellipse, and *a* is the distance from the center to a vertex of an ellipse; Fig. 1C) with their major axis aligned near‐parallel to the contractile filament bundles (Fig. 1D). During contracture, mitochondria become more compact and turn into less regular‐shaped volumes, often akin to convex polyhedrons (Supporting Information Fig. S1A), with their main axis orientation becoming more variable. In parallel to these changes in mitochondrial shape, the interfibrillar gap width (measured at the level of M‐lines of neighboring sarcomeres) becomes larger in contracture, compared to rest and stretch (Supporting Information Fig. S1B).

### Microtubular Cytoskeleton and Mitochondrial Arrangement

**Figure 2:**
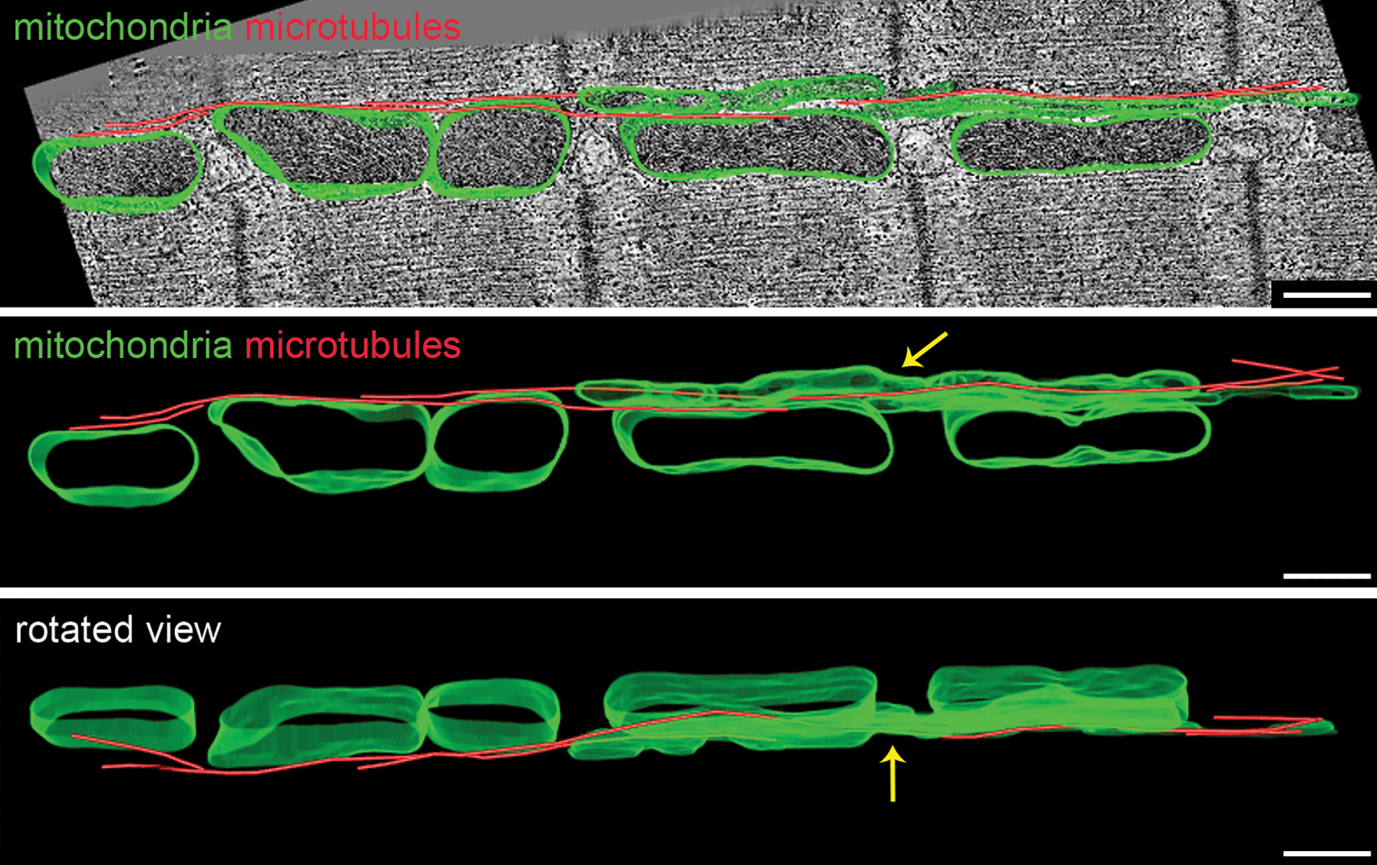
Microtubular network and interfibrillar mitochondria. Representative electron tomographic micrographs with 3D segmented models overlaid (top panel), separate (middle panel) and displayed at different rotation angle (bottom panel; mitochondria‐green, microtubules‐red). Microtubules run along the intersarcomeric space and interweave with several mitochondria in a row. Note that some mitochondria have an elongated shape spanning more than one sarcomere (arrow). Scale bar: 500 nm.

**Figure 3:**
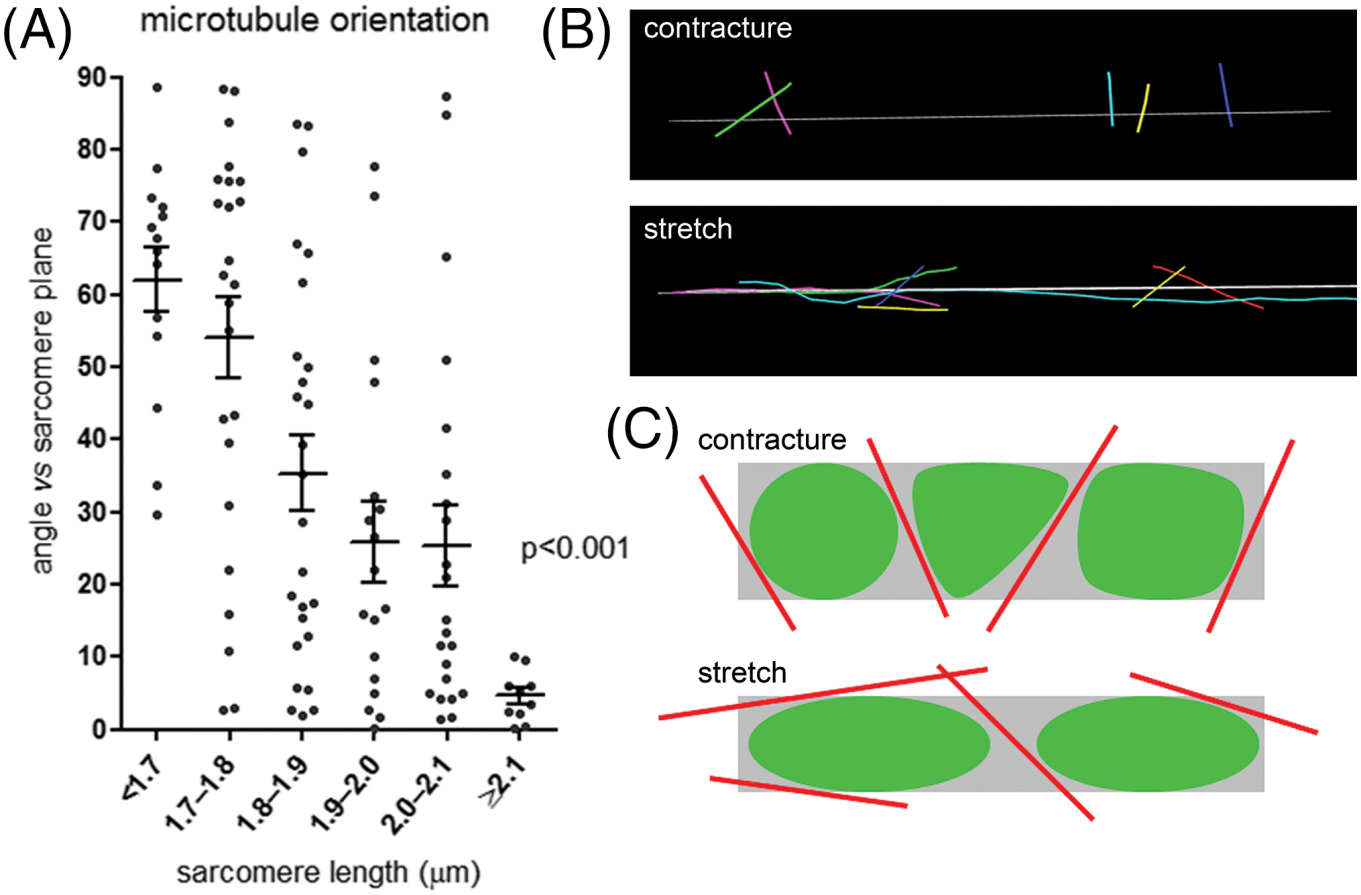
The orientation of microtubules changes with the sarcomere length, and correlates with mitochondrial deformation. (**A**) The minimum intersection angle between microtubules and the associated sarcomere plane decreases with increasing sarcomere length, n = 14 to 26 microtubules per group, N = 6 animals, analysis with one‐way ANOVA. (**B**) Representative 3D segmented models of microtubules in cells fixed in different deformation states; gray line illustrates axial orientation of associated sarcomeres. (**C**) Schematic of the correlation of microtubular orientation and mitochondrial deformation in different mechanical states.

Careful examination of interfibrillar mitochondrial networks in 3D electron tomographic stacks revealed an abundant presence of interweaved microtubules (Fig. 2), running along the interfibrillar space. Analysis of the angle of relative to the direction of contractile filaments microtubules in different mechanical states revealed that this angle is higher during contracture compared to stretched cells (Fig. 3), consistent with the 3D deformation of individual mitochondria described in Figure 1 and Supporting Information Figure S1.

### Mitochondria Form a Structurally Integrated Network

**Figure 4:**
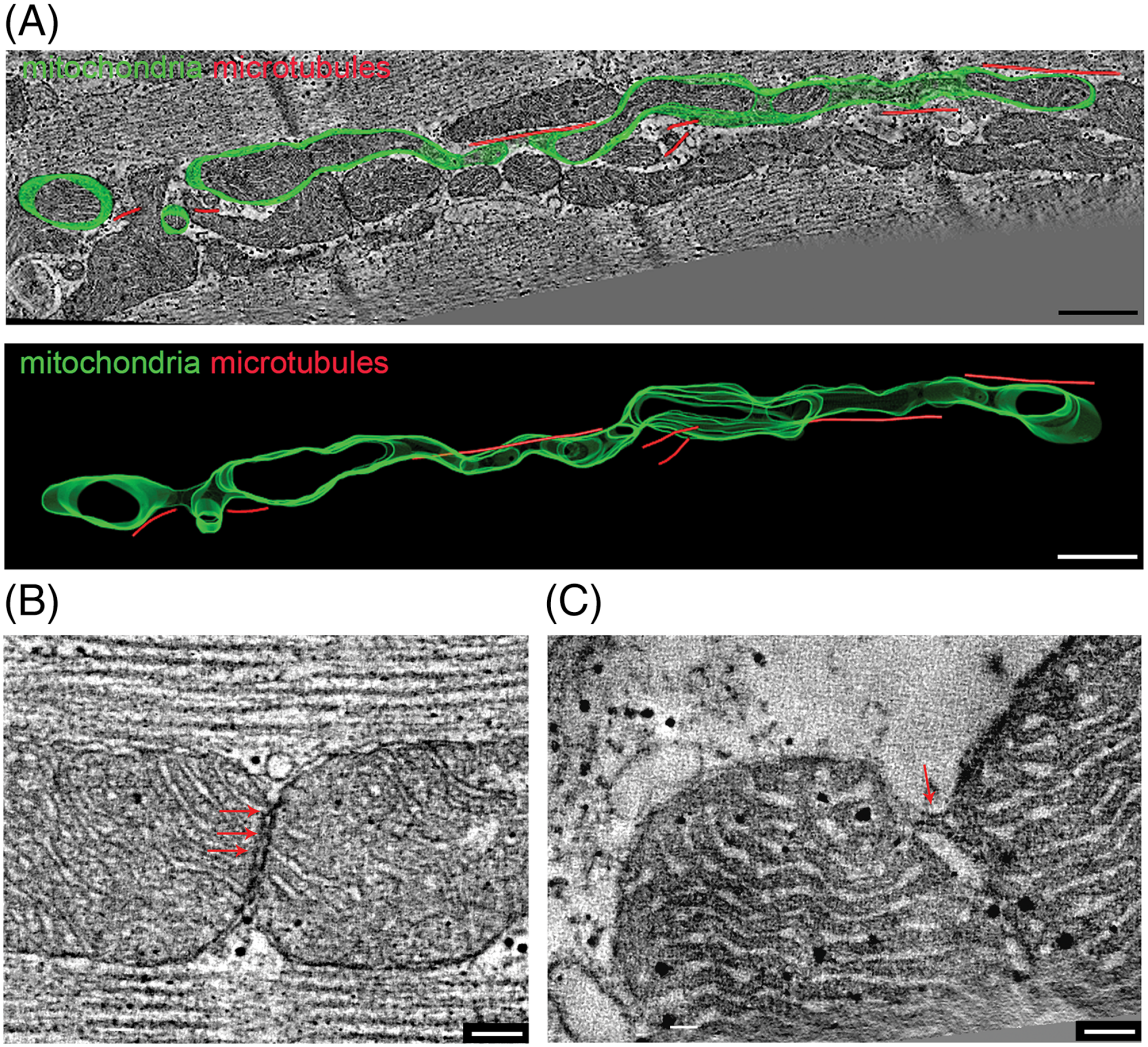
Mitochondria form locally interconnected 3D networks extending beyond a single sarcomere in rabbit ventricular cardiomyocytes. (**A**) Representative electron tomographic micrograph and 3D segmented model of a single mitochondrion (green) and associated microtubules (red), showing an elongated tortuous 3D arrangement that is not evident from 2D sections alone (cf., EM section, top, and 3D reconstruction, bottom). In addition, ultrastructural tethers (**B**) and membranous bridges (**C**) are present between seemingly separate mitochondria (visualized by suitable selection of 2D cutting planes through the 3D tomography data sets). Scale bars: 500 nm (in A) and 100 nm (in B and C).

While 2D sections tend to show mitochondria as apparently disconnected ellipsoids, 3D reconstruction reveals the presence of individual long‐winded, tortuous mitochondrial structures (Fig. 4A). Additionally, otherwise separate mitochondria can be linked by structural tethers (Fig. 4B), as well as membranous bridges (Fig. 4C), all supporting the notion of long‐distance communication inside the mitochondrial network (Glancy et al., 2017).

### Mitochondria Form Structural Tethers With Sarcoplasmic Reticulum and T‐Tubules

**Figure 5:**
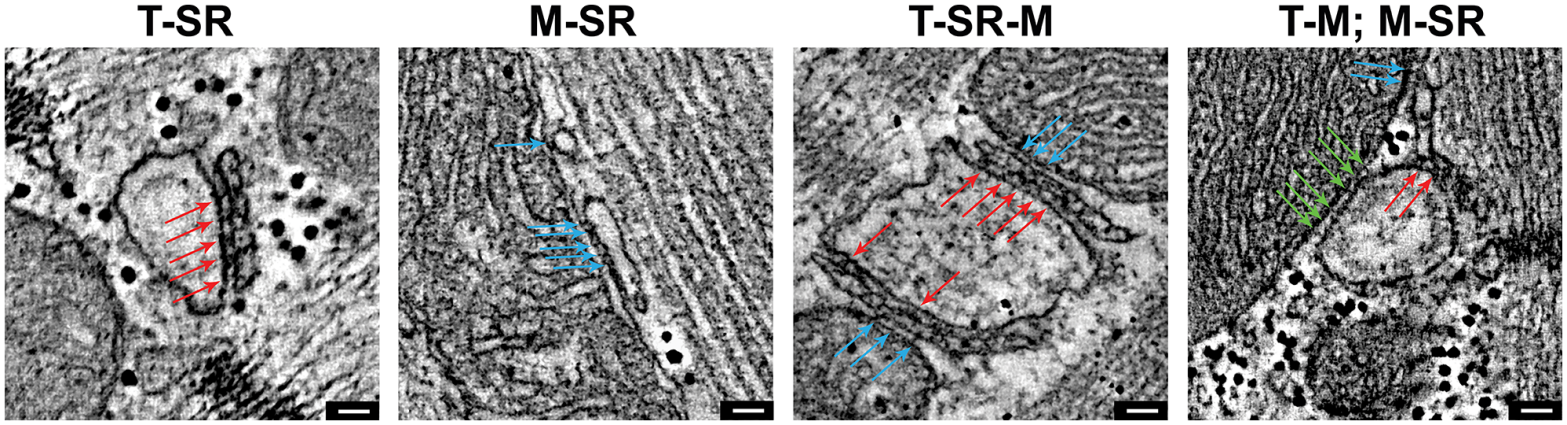
Electron‐dense structures between mitochondria (M) and both junctional and network sarcoplasmic reticulum (SR) as well as T‐tubules (T). Representative electron tomographic micrographs highlighting the presence and similarity of electron‐dense structures connecting T‐tubules and SR (red arrows: ryanodine receptors), SR and mitochondria (blue arrows) and T‐tubules and mitochondria (green arrows). Scale bars: 50 nm.

Consistent with previous studies (Hayashi et al., 2009), mitochondria were found to form electron‐dense, often regularly spaced tethers with sarcoplasmic reticulum (SR) cisterns, including both T‐tubule associated junctional, as well as network SR (Fig. 5). Structural connections were also observed between T‐tubules and mitochondria (Fig. Error! Reference source not found.), often creating a continuous “multi‐structure” of T‐tubule SR and mitochondria. The size of these electron‐dense entities was not different from that of ryanodine receptors (RyR), seen regularly between T‐tub and SR in the same samples. The maximum width of electron densities at the interface of T‐tubule and SR was 14.6 nm [±2.4 nm (standard deviation, SD), n = 92, N = 6 animals]; at the interface between SR and mitochondria 13.7 nm (±3.2 SD, n = 106, N = 6 animals); and between T‐tubules and mitochondria 13.9 nm (±2.7 SD, n = 104, N = 6 animals). The interdensity distances at the interface of T‐Tubule and SR were 17.33 nm (± 3.6 SD, n = 107, N = 6 animals); at the interface between SR and mitochondria 17.76 nm (± 4.14 SD, n = 104, N = 6 animals); and between T‐tubules and mitochondria 18.05 nm (± 5.03 SD, n = 94, N = 6 animals)

## DISCUSSION

First, mitochondrial shape changes correlate with the mechanical state of the cell: with increasing sarcomere length, interfilament gap width becomes progressively narrower, and interfibrillar mitochondria become more elongated in a near‐parallel direction to contractile filaments (see Fig. 1). Our findings extend previous light‐microscopy based information (Yaniv et al., 2011), with higher resolution 3D data that, in addition to slack and contracted cells, includes sarcomere stretch to levels that cells will experience during diastolic loading. With stretch, there is a progressive change of cross‐sectional shapes from relatively compact polygons toward elongated elliptoids. The significance and functional consequences of these shape changes will require further research; they could potentially contribute to mitochondrial mechanosensing and mechanotransduction. Cardiac mitochondria—both in cardiomyocytes and cardiac fibroblasts—respond to deformation with changes in their membrane potential, ATP/ ROS production, and activation of cell cycle and apoptotic pathways (Liao et al., 2004; Iribe et al., 2017). In addition, mitochondria are capable of accumulating Ca^2+^ and may buffer cytosolic Ca^2+^ fluctuations (Dedkova & Blatter, 2008), potentially in a mechanically modulated manner (Morad et al., 2005; Belmonte & Morad, 2008; Iribe et al., 2009; Miragoli et al., 2016). A potentially related phenomenon, mitochondrial swelling, may also influence cardiac force development, contractility, and even gene expression, through changes in internal pressure and by affecting the morphology of neighboring organelles (Kaasik et al., 2010; Burton et al., 2017).

Second, mechanical effects on mitochondria may be mediated not only via beat‐by‐beat “lateral squeezing” by neighboring contractile lattice structures (Yaniv et al., 2011), but also through mechanical cues from cytoskeletal elements (Saetersdal et al., 1990). In eukaryotes, this interaction is primarily mediated by microtubules, which have been shown to physically bind to the outer mitochondrial membrane (Hirokawa, 1982; Anesti & Scorrano, 2006; Kuznetsov et al., 2013). Mitochondria often display a subcellular distribution corresponding to that of the cytoplasmic microtubular network (Heggeness et al., 1978), and disruption of microtubular integrity can lead to altered mitochondrial distribution (Miragoli et al., 2016). This may be relevant for mitochondrial function, which is additionally affected by properties of the extracellular matrix such as elasticity and fiber alignment (Lyra‐Leite et al., 2017). Here, we confirm the close spatial interrelation between mitochondrial and microtubular networks, and the presence of matching spatial rearrangement of the two in the context of changes in sarcomere length (Figs. 2 and 3).

Third, cell deformation alters the complex 3D arrangement of the mitochondrial network. It is important to note that 3D structures may not always be inferred successfully from 2D section data. This should be obvious for any 2D mitochondrial shape that is incompatible with rotational symmetry. But even when cross‐sections are (near‐)elliptical, it does not necessarily follow that the associated organelle forms a prolate spheroid. In fact, elongated “snake‐like” configurations are present in healthy myocardium (Fig. 4A). These may be more frequent in metabolic diseases (Tsushima et al., 2018). Of note, deformation of such complex 3D structures would not be associated with the stretch‐induced mismatch in volume:surface ratio one would encounter in spheroids.

Fourth, our data corroborates the view that individual mitochondria do not function in isolation from one another, but constitute a dynamic network of communicating organelles (Glancy et al., 2017) http://science.sciencemag.org/content/361/6401/eaan5835/tab-article-info. Mitochondrial shape in ventricular myocytes was highly variable, not only between but also within individual cells (e.g., Fig. 2). As mentioned, aside from “classic” ellipsoids, our 3D data revealed the presence of non‐ellipsoid convex polyhedrons—in particular at short sarcomere lengths—and elongated snake‐like mitochondria. Any 2D cut through the latter may give rise to multiple, apparently independent sections of the same mitochondrion, including “elliptic” sections that could erroneously be interpreted as multiple individual spheroid‐shaped organelles. We also observed electron‐dense intermitochondrial connections and membranous nano‐junctions between otherwise separate mitochondria (Fig. 4B and C), consistent with previous reports of the existence of a “mitochondrial reticulum” (Glancy et al., 2015). The presence of such contacts has been observed before (Huang et al., 2013; Lavorato et al., 2017), and they are thought to enable mitochondrial communication in cells with restricted mitochondrial motility (such as muscle cells), for example during periods of stress (Vincent et al., 2017). Additionally, a structural/functional syncytium has been hypothesized to enable tight, rapid electrical coupling between mitochondria, with the mitochondrial network proposed to work as cell‐wide coupled oscillator, capable of receiving and transmitting mechanical stimuli (Aon et al., 2006). Currently, it is not known which mitochondrial morphology (individual spheroids or contorted tubules) is closer to the native state *in vivo*, and whether/how geometric features of organelles change during sample preparation (here, we took care to use isosmotic solutions and to complete sample fixation in under 10 min from organ excision and exposure to crystalloid solutions). The mitochondrial network is thought to be highly dynamic, including fusion and fission of mitochondria. Mitochondrial morphology can change dramatically in response stress (Mishra & Chan, 2014). Cells with a high fusion‐to‐fission ratio may have a predominance of long, tubular mitochondria forming a highly interconnected network; conversely, cells with a low fusion‐to‐fission ratio may mainly have fragmented mitochondria with reduced connectivity (Sesaki & Jensen, 1999). Additionally, mitochondrial tubulation has been proposed as a mechanism for creation of dynamic thin tubules, and eventually lattices that interconnect to generate the mitochondrial network (Wang et al., 2015).

Fifth, mechanical deformation of mitochondria can affect cardiomyocyte function through interaction with the SR (Franzini‐Armstrong, 2007). Mitochondria have previously been shown to be physically tethered to the SR in mouse myocardium (Hayashi et al., 2009), and similar structures are reported here for rabbit ventricular cardiomyocytes (Fig. 5). The close proximity of the two compartments is thought to play a role in Ca^2+^ signaling, exchange, and buffering and, consequently, to modulate excitation‐contraction coupling (Duchen et al., 1998; Rizzuto et al., 1998; Lu et al., 2013). SR‐mitochondrial Ca^2+^ transfer is believed to occur through direct physical contact in cardiomyocytes (Lopez‐Crisosto et al., 2017), and the molecular identity of those tethers has been previously suggested as mitofusin (Naon et al., 2016). Some of the structures described here share structural similarity to mitochondrial ryanodine receptors, as proposed in earlier studies (Csordás et al., 2001; Franzini‐Armstrong, 2007; Lukyanenko et al., 2007). The consequences of mitochondrial deformation for structural cross‐talk with the SR are not known. Previously, cytoarchitectural perturbations have been shown to affect SR‐mitochondrial functional coupling (Wilding et al., 2006; Joubert et al., 2008; Lopez‐Crisosto et al., 2017). Additionally, we describe electron dense RyR‐like structures between T‐tubules and mitochondria.

The insight described under points one to five needs to be considered in the context of several study limitations. These include the lack of same‐cell observations and true beat‐by‐beat temporal resolution of the sample processing approach (reaching the desired mechanical state required minutes, not seconds). Both limitations are difficult to address while acquiring 3D nano‐scale data from cells *in situ*. We cannot confirm, therefore, whether the changes we describe here will also occur on a beat‐by‐beat basis (although a previous study, using single‐axis 10‐nm‐resolution line scan analysis, would seem to support such an extrapolation; see (Yaniv et al., 2011)).

Overall, we conclude that mitochondria become more elongated in a sarcomere‐parallel direction as sarcomere length increases from peak‐contraction to diastolic stretch. This elongation is associated with a principal change in mitochondrial shape, from convex polyhedrons to prolate spheroids, alleviating the otherwise present mismatch in volume‐surface ratio. Individual mitochondria can from elongated tube‐like structures that extend over multiple sarcomeres and have a length‐to‐width ratio exceeding 30:1. We see evidence of outer membrane connections of neighboring mitochondria, and of (RyR‐sized and ‐spaced) electron‐dense structural connections of mitochondria with the SR and T‐tubules. In this context, 3D nano‐scale reconstructions are a vital enabling methodology, both to avoid errors in projecting from 2D section data to 3D structural properties (mitochondrial “snakes”), and to select virtual cutting planes that allow characterization of rare (intermitochondrial membrane tethers), punctate (RyR), or high aspect ratio elongated structures (microtubules).

## Supporting information

Supplementary Figure 1. Mitochondrial shape and interfilament gap width change with mechanical state of cardiomyocytes. (A) Contribution (% of total mitochondria) of various mitochondrial shapes at different sarcomere lengths. Three mitochondrial shapes were distinguished (1–‐3), with major (*x*) and minor (*y*) axis relationship as follows: 1) structures where *x* is perpendicular *y* and the two diameters intersect each other more or less in their respective midpoints (*e.g*. an elliptoid); 2) structures where *x* is perpendicular *y* but the two intersect each other in a place that is not their respective midpoints (*e.g*. a triangle); 3) structures where *x* is not perpendicular *y* (*e.g*. non‐equilateral parallelograms)*;* n = 42 to 83 mitochondria per group, N = 6 animals (B) Interfilament gap width (as measured between two neighbouringneighboring M‐lines) is highest during contracture; n = 10 to 24 gaps measured per group, N = 6 animals, p < 0.001, analysis with one‐way ANOVA.
