# Supplementary figures and images for: Mitochondrial Deformation During the Cardiac Mechanical Cycle
Source: Anat Rec (Hoboken). 2018 Oct 10;302(1):146–52. doi: 10.1002/ar.23917 (PMC6312496; doi:10.1002/ar.23917)

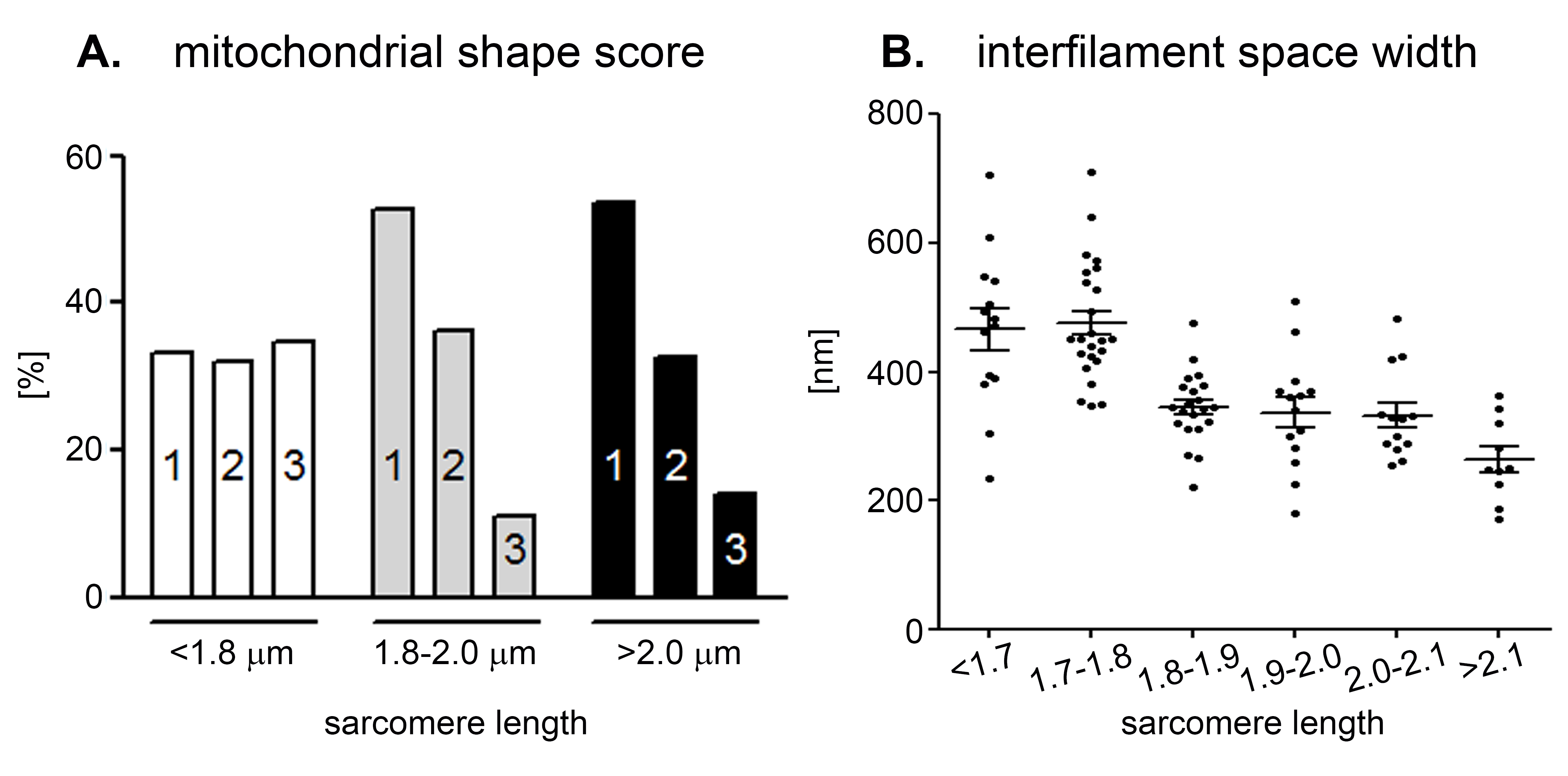

Supplement: Supplementary file 1 — Supplementary Figure 1. Mitochondrial shape and interfilament gap width change with mechanical state of cardiomyocytes. (A) Contribution (% of total mitochondria) of various mitochondrial shapes at different sarcomere lengths. Three mitochondrial shapes were distinguished (1–‐3), with major (x) and minor (y) axis relationship as follows: 1) structures where x is perpendicular y and the two diameters intersect each other more or less in their respective midpoints (e.g. an elliptoid); 2) structures where x is perpendicular y but the two intersect each other in a place that is not their respective midpoints (e.g. a triangle); 3) structures where x is not perpendicular y (e.g. non‐equilateral parallelograms); n = 42 to 83 mitochondria per group, N = 6 animals (B) Interfilament gap width (as measured between two neighbouringneighboring M‐lines) is highest during contracture; n = 10 to 24 gaps measured per group, N = 6 animals, p < 0.001, analysis with one‐way ANOVA. [file AR-302-146-s001.tif]
